# Translational applicability of human blood-brain barrier spheroid models for the development of brain-penetrant therapeutic antibodies

**DOI:** 10.1186/s12987-025-00752-8

**Published:** 2026-01-24

**Authors:** Seiya Ohki, Tomoki Fukatsu, Hideto Morimoto, Masafumi Kinoshita, Atsushi Imakiire, Ryuji Yamamoto, Hanae Morio, Hiroyuki Sonoda, Tomomi Furihata

**Affiliations:** 1 Laboratory of Advanced Drug Development Sciences, School of Pharmacy, Tokyo University of Pharmacy and Life Sciences, 1432-1 Horinouchi, Hachioji, Tokyo, 192-0392 Japan; 2 JCR Pharmaceuticals, Research Division, Kobe, Hyogo 659-0021 Japan

**Keywords:** Blood‒brain barrier, Microphysiological system, In vitro-in vivo correlation, Anti-transferrin receptor antibody, Drug delivery system

## Abstract

Drug delivery systems play a key role in the development of new therapeutic strategies for brain diseases. Antibodies capable of crossing the blood–brain barrier (BBB) have attracted considerable attention as promising drug carriers. To facilitate their development, we previously established human multi-cellular spheroidal (hMCS)-BBB models as microphysiological system-based platforms. In this study, we aimed to enhance the applicability of these hMCS-BBB models for the development of brain-permeable antibodies using four anti-human transferrin receptor monoclonal antibodies (hTfRMAbs). Permeability assays using hMCS-BBB models revealed BBB permeability in three out of four hTfRMAbs, with two antibodies exhibiting *Km* values of 6.5 and 12.0 µg/mL, respectively. Importantly, differences in the BBB permeability of tested hTfRMAbs were consistent with the brain permeability profiles reported in monkeys in vivo. Moreover, these differences were not detected via simple uptake assays using monoculture brain microvascular endothelial cells, highlighting the superior evaluation potential of hMCS-BBB models. Lysosome co-localization assays using hMCS-BBB models revealed the distinct intracellular localization patterns of BBB-permeable and non-permeable hTfRMAbs with lysosomes, presumably explaining their different permeability properties. Overall, hMCS-BBB models effectively characterized the BBB permeability of hTfRMAbs, demonstrated strong potential for in vivo extrapolation, and proved suitable for mechanistic studies of the cellular pathways regulating their BBB permeability. Therefore, hMCS-BBB models are considered promising tools for advancing the development of brain-permeable therapeutic antibodies.

**Graphical Abstract:**

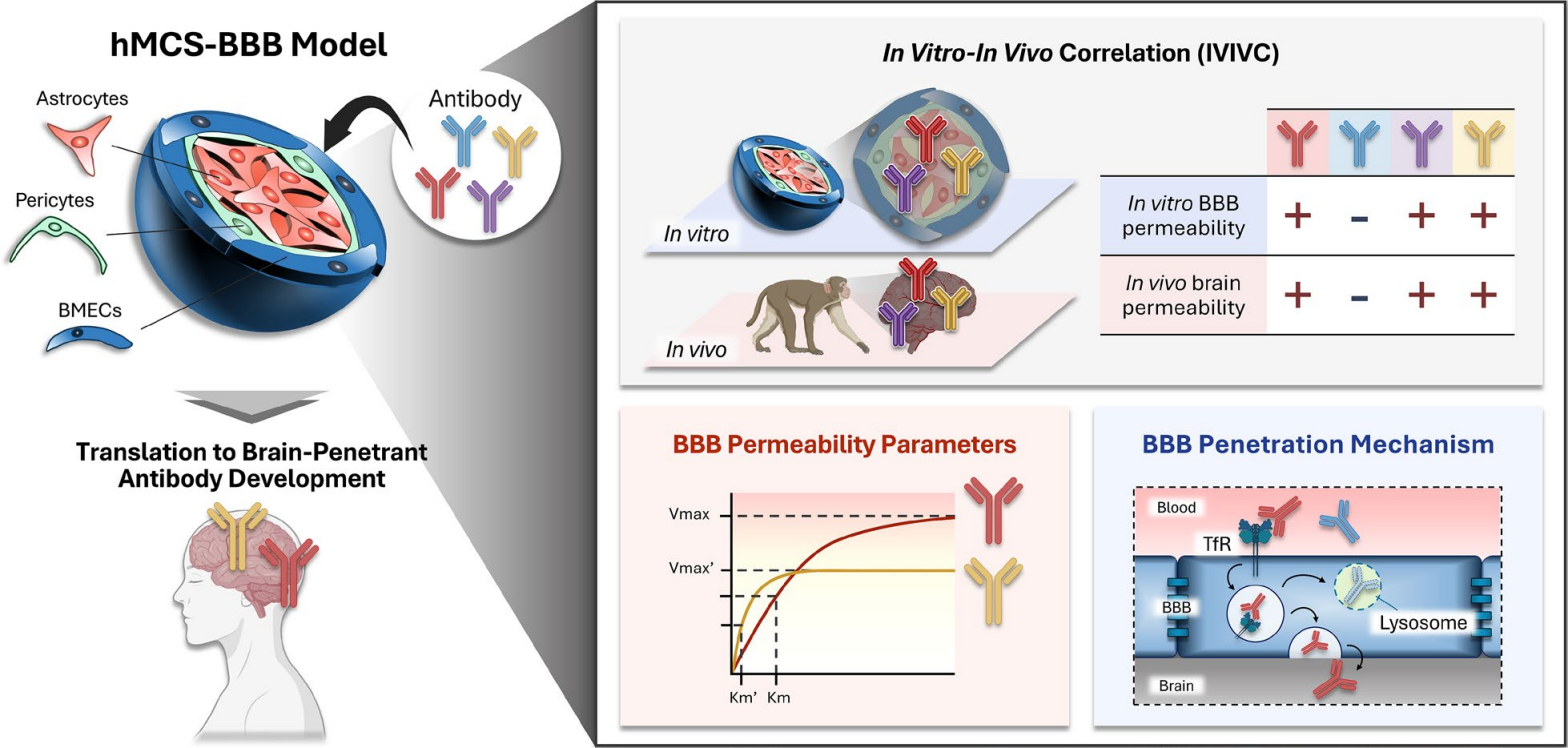

**Supplementary Information:**

The online version contains supplementary material available at 10.1186/s12987-025-00752-8.

## Introduction

The blood‒brain barrier (BBB) is primarily composed of brain microvascular endothelial cells (BMECs), which form tight junctions (e.g., zona occludens-1 [ZO-1] and claudin-5) and adherens junctions (e.g., vascular endothelial [VE]-cadherin and β-catenin) and express unique influx and efflux transport systems that regulate the entry of various substances into the brain [1, 2]. These structural and functional properties are known to be supported by surrounding astrocytes and pericytes [1, 2]. As one of the most selective barriers in the body [3, 4], the BBB is a major obstacle to brain drug delivery. Not only small molecules but also biologics/macromolecules exhibit low permeability across the BBB; for example, IgG reaches the brain at extremely low levels - only approximately 0.25% of the administered dose [4, 5].

Recently, biologics have attracted considerable attention as potential therapeutics for central nervous system diseases. To achieve therapeutic concentrations in the brain, biologics require brain-permeable drug delivery systems (DDSs) for enhanced BBB permeability. Specifically, antibody-based brain DDSs show great potential due to their high target specificity and long systemic half-life. Brain-permeable antibodies enter the brain via receptor-mediated transcytosis (RMT) across the BBB [6, 7]. RMT generally involves three steps: Antibody binds to a receptor on the luminal membrane of BMECs and is internalized via endocytosis, after which the receptor–antibody complex moves toward the opposite side via vesicular transport, and the antibody is released into the brain via exocytosis [6, 7]. Transferrin receptor (TfR) is one of the most extensively investigated RMT receptors, and a successful example of a brain-penetrant biologic utilizing TfR is pabinafusp alfa, a fusion protein of iduronate-2-sulfatase and an anti-human TfR monoclonal antibody (hTfRMAb), the latter of which serves as a brain DDS [8]. By leveraging TfR-mediated transcytosis, pabinafusp alfa exhibits clinical efficacy in ameliorating the central nervous system symptoms of mucopolysaccharidosis II [9, 10]. Another example is trontinemab, which carries a single-domain Fab targeting TfR, and is currently under clinical investigation for anti-amyloid-β antibody delivery to treat Alzheimer’s disease [11].

Accurate prediction of human brain permeability and characterization of individual BBB permeability profiles during preclinical stages are crucial to develop DDSs with high efficiency. However, interspecies differences in BBB between humans and experimental animals pose critical limitations [12–14]. Consequently, data obtained using animal models cannot always be extrapolated to humans, increasing the difficulty of predicting the brain permeability of antibodies.

One promising method to address the above-mentioned issue is the use of in vitro human BBB models. Particularly, microphysiological system (MPS)-based human BBB models have attracted significant attention [15–17]. MPSs are sophisticated in vitro heterogeneous cell co-culture systems designed to replicate the in vivo microenvironment by integrating various physiological factors, thereby enhancing the inherent cellular functions. Recently, we have developed a human multi-cellular spheroidal (hMCS)-BBB model as a representative MPS-BBB model [18, 19]. Our hMCS-BBB model exhibits a three-dimensional architecture comprising an outer layer of human BMEC/conditionally immortalized clone 18 (HBMEC/ci18 [20]) forming the BBB, which surrounds an internal core composed of human brain pericytes/conditionally immortalized clone 37 (HBPC/ci37 [21]) and human astrocytes/conditionally immortalized clone 35 (HASTR/ci35 [22, 23]). We have also shown that hMCS-BBB models can be used to evaluate the BBB permeability differences between anti-TfR antibodies (MEM189) and naïve IgG [19].

Further evaluation of their potential is necessary to effectively utilize hMCS-BBB models for human brain-penetrant antibody development. Specifically, the validity and scope of these models in evaluating the BBB permeability of antibodies remain to be fully elucidated. Therefore, in this study, we evaluated the in vitro-in vivo BBB permeability correlations (IVIVC) of four hTfRMAbs and examined their kinetic BBB permeability profiles to enhance the applicability of hMCS-BBB models for brain-permeable antibody development. Furthermore, we sought to gain mechanistic insight into the observed differences in the BBB permeability of the tested hTfRMAbs.

## Materials and methods

### Cells and hMCS-BBB model development

HBMEC/ci18, HASTR/ci35, and HBPC/ci37 cells were cultured as described in our previous studies [20–23]. Characteristics of each immortalized cell line are also described therein.

hMCS-BBB models were developed as previously described [19, 24]. Briefly, the models were prepared by sequentially seeding the three cell types in 96-well V-bottom plates (Sumitomo Bakelite, Tokyo, Japan) in the following order: HASTR/ci35 cells (1,750 cells/well) on day 1, HBPC/ci37 cells (500 cells/well) on day 2, and HBMEC/ci18 cells (750 cells/well) on day 3. The models were used for assays on day 5. All procedures were performed at 37 °C using the VascuLife VEGF Comp kit (Lifeline Cell Technology, Frederick, MD, USA) supplemented with penicillin–streptomycin (Nacalai Tesque, Kyoto, Japan) and 0.48 mg/mL methylcellulose (Wako, Osaka, Japan).

### Generation of HBMEC/ci18^TfR − KO^ cells

Single guide RNA (sgRNA) described by Simonneau et al. [25] (PAM sequence: CGG; DNA target sequence: TGATCATAGTTGATAAGAACGG) was used for the *TfR* gene knockout (TfR-KO). HBMEC/ci18 cells were transduced with lentiviral particles carrying both sgRNA and clustered regularly interspaced palindromic repeat-associated protein 9 (CRISPR-Cas9; Vector Builder Inc., Chicago, IL, USA). TfR-KO HBMEC/ci18 cells (HBMEC/ci18^TfR − KO^) were selected with puromycin (Nacalai Tesque) at 0.3 µg/mL for seven days. These cells were individualized using the previously described limiting dilution method [26] to isolate several HBMEC/ci18^TfR − KO^ clones.

### Western blotting analysis

Whole-cell lysates of HBMEC/ci18 and HBMEC/ci18^TfR − KO^ cells cultured in VEGF- and hEGF-free (V/E-free) VascuLife medium at 37 °C for 24 h were prepared using the RIPA lysis buffer (Nacalai Tesque). Protein concentrations in the lysates were determined using a BCA protein assay kit (Takara Bio, Shiga, Japan). Subsequently, a protease inhibitor cocktail (Nacalai Tesque) was added to the samples. For Western blotting, lysates containing 0.5 µg protein each were separated using ePAGEL HR gel (12.5%; ATTO, Tokyo, Japan) and transferred to polyvinylidene fluoride membranes (Bio-Rad Laboratories, Hercules, CA, USA). The membranes were blocked overnight with 4% (w/v) Block ACE (KAC, Kyoto, Japan). All primary and secondary antibodies used here are listed in Table S1. All antibodies were diluted with Signal Enhancer HIKARI for Western Blotting and enzyme-linked immunosorbent assays (ELISA) (Nacalai Tesque), and the cells were incubated with these antibodies for 1 h each. Finally, immunocomplexes were detected using LAS-3000 (Fujifilm, Tokyo, Japan).

### Immunocytochemistry (ICC)

Approximately ten spheroids (hMCS-BBB models) were pooled in a Proteosave SS 0.5-mL microtube (Sumitomo Bakelite) and washed with phosphate-buffered saline (PBS(–); Nacalai Tesque), after which ICC of barrier-associated proteins (ZO-1, VE-cadherin, and β-catenin) was performed as previously described [19]. All primary and secondary antibodies used for ICC are listed in Table S1. All antibodies were diluted to the indicated concentrations using the Can Get Signal Immunostain Solution A (TOYOBO, Osaka, Japan). The spheroids were mounted on glass slides, and the nuclei were stained with 4’,6-diamidino-2-phenylindole (DAPI; Nacalai Tesque). Fluorescence was detected using the FLUOVIEW FV3000 confocal microscope (Evident, Tokyo, Japan). Microscopic parameters, such as laser intensity, were kept constant during all comparative fluorescence analyses.

### Tf uptake assays using monocultured HBMEC/ci18 cells

HBMEC/ci18 or HBMEC/ci18^TfR − KO^ cells (3.0 × 10^4^ cells/well) were cultured in V/E-free VascuLife for 24 h in 12-well plates at 37 °C. The cells were incubated at 37 °C with Alexa Fluor 647-conjugated Tf (500 nM; Jackson Immuno Research, West Grove, PA, USA) in the human endothelial serum-free medium (SFM; Thermo Fisher Scientific, Waltham, MA, USA) for 20 min. Then, the cells were collected using TrypLE (Thermo Fisher Scientific) and incubated on ice until analysis. After filtration through a cell strainer (Corning Life Sciences, BD Falcon, Franklin Lakes, NJ, USA), fluorescence intensity of each cell was monitored using LSRFortessa X-20 (BD Biosciences, Milpitas, CA, USA). The mean fluorescence intensity was analyzed using the FACSDiva software (BD Biosciences).

### Lucifer yellow (LY) permeability assays using hMCS-BBB models

LY permeability assays using hMCS-BBB models were performed as previously described [19, 27]. In addition to hMCS-BBB models, spheroids without HBMEC/ci18 cells (ΔBMEC models) were prepared for comparison. Briefly, approximately ten spheroids for each model were pooled in a 0.5-mL microtube, washed once with the Hanks’ balanced salt solution with calcium and magnesium (HBSS+; Nacalai Tesque), and incubated with HBSS(+) containing LY (50 µM; Wako) for 90 min at 37 °C. The spheroids were washed twice with PBS(+), fixed with 4% paraformaldehyde (Nacalai Tesque) for 10 min at room temperature, and mounted on glass slides. Intracellular fluorescence was immediately assessed using the FV3000 confocal microscope at a depth of 50 μm, and average fluorescence intensity per area was quantified using the FV31S-SW software (Evident).

### hTfRMAbs

Four types of hTfRMAbs (hTfRMAb-1, -2, -3, and − 4) were prepared by JCR Pharmaceuticals (Kobe, Japan). All hTfRMAbs are in a bivalent mouse IgG format. In vivo brain permeability profiles of these hTfRMAbs have been previously characterized using cynomolgus monkeys [28]. Briefly, hTfRMAb-1, -3, and -4 exhibit clear distribution throughout the brain parenchyma and are therefore classified as BBB-permeable, whereas hTfRMAb-2 localizes primarily to the brain vascular endothelium without parenchymal penetration and is classified as non-permeable. Detailed in vivo data for the hTfRMAbs, including their brain concentrations and representative immunohistochemical images after i.v. administration, are provided in the (Supplemental materials and methods, Table S2, and Fig. S1).

### Binding properties of hTfRMAbs to human TfR

The K_D_ values for the binding of each hTfRMAb to human TfR were determined using bio-layer interferometry with the Octet RED96 system (Pall ForteBio, Fremont, CA), as previously reported [8]. Briefly, each hTfRMAb at various concentrations (0–40 nM) was applied to the recombinant hTfR extracellular domain immobilized on Ni-NTA biosensors. Kinetic constants were calculated from the sensorgrams using the 1:1 Langmuir binding model.

The EC_50_ values for hTfRMAb binding to human TfR were determined by ELISA. Nunc-Immuno MicroWell 96-well plates (Thermo Fisher Scientific) were coated with in-house recombinant human TfR (hTfR; 5 µg/mL in 50 mM NaHCO_3_, pH 9.6) for 1 h at room temperature. After washing with PBS with Tween (PBS-T), hTfRMAbs were serially diluted in three steps with 0.1% bovine serum albumin (BSA)/PBS-T (ranging from 0.69 to 500 ng/mL), added to each well and incubated for 1 h at room temperature. Following PBS-T washes, anti-mouse IgG (H + L) HRP-conjugated secondary antibodies (Bethyl Laboratories, Montgomery, TX, USA) were added and incubated for 30 min at room temperature. After washing with PBS-T, the 3,3’,5,5’-tetramethylbenzidine (TMB) substrate solution was added and incubated for 5 min. The reaction was stopped with 1 M H_2_SO_4_ (Wako), and the absorbance was measured at 450 nm using a microplate reader. The EC_50_ values were calculated from the concentration-response curves.

### Antibody permeability assays using hMCS-BBB models

Permeability assays for hTfRMAb using hMCS-BBB models were performed as previously described [19]. Four types of hTfRMAbs were prepared by JCR Pharmaceuticals as described above (Hyogo, Japan). Isotype control IgG was obtained from ProteinTech (Rosemont, IL, USA) and used as a non-specific control. All antibodies were fluorescently labeled with HiLyte Fluor 647-Labeling Kit-NH_2_ (Dojindo Molecular Technologies, Kumamoto, Japan). Note that labeling the antibodies did not affect their affinity for hTfR as determined by the above-described ELISA (Table S3).

hMCS-BBB models were incubated with each antibody a final concentration of 2 µg/mL in SFM containing iron-saturated holo-Tf (Wako) for 2 h at 37 °C or 4 °C. The spheroids were collected and mounted on glass slides, and their fluorescence intensities were quantified as described above. Signal intensity was determined by subtracting the values of the corresponding blank spheroids (background) from those of the test spheroids. Uptake activity of hTfRMAbs was expressed as the ratio of the intraspheroidal fluorescence intensities at 37 °C and 4 °C (37/4°C).

### Characterization of the BBB permeability properties of brain-penetrating hTfRMAbs

For time-dependent analysis, the spheroids were incubated with hTfRMAb-1 or -4 (2 µg/mL each in SFM containing iron-saturated holo-Tf) for 0.5, 1, 2, 4, and 6 h at 37 °C or 4 °C, after which the fluorescence intensities were detected as described above. The resulting signal intensities at each time point were plotted, and a fitting curve was drawn using the Prism8 software (MDF, Tokyo, Japan). Time-dependent uptake profile was generated by subtracting the fluorescence intensities at 4 °C from those at 37 °C (dotted line, Δ4°C) to obtain the linear uptake range.

For concentration-dependent analysis, control or TfR-KO models were incubated with hTfRMAb-1 or -4 (1, 2, 5, 10, and 20 µg/mL each in SFM containing iron-saturated holo-Tf) for 2 h or 1 h, respectively, at 37 °C. TfR-dependent uptake profile was generated by subtracting the fluorescence intensity of TfR-KO models from that of the control models (dotted line, ΔTfR) to obtain the *Km*_*TfR*_ values.

### hTfRMAb uptake assays using monocultured HBMEC/ci18 cells

HBMEC/ci18 and HBMEC/ci18^TfR − KO^ cells (3.0 × 10^4^ cells/well) were cultured in V/E-free VascuLife for 24 h in 12-well plates at 37 °C. The cells were incubated with hTfRMAbs or IgG (1.0 µg/mL each in SFM containing iron-saturated holo-Tf) for 20 min at 37 °C and 4°C. The cells were washed quickly with PBS(–) and lysed using the RIPA lysis buffer. The supernatant was collected via centrifugation (10,000 × *g*, 10 min, 4°C). Total protein concentration in each lysate was determined using the BCA protein assay kit, and fluorescence intensities of hTfRMAbs were quantified using a microplate reader (BioTek, Winooski, VT, USA).

### Co-localization analysis of hTfRMAbs with lysosomes in hMCS-BBB models

The spheroids collected in a tube were incubated with LysoTracker (200 nM in HBSS(+); Thermo Fisher Scientific) at 37 °C for 60 min for lysosome staining. After washing with PBS(+), the spheroids were pre-incubated in SFM containing 25 µM iron-saturated holo-Tf for 30 min. Subsequently, they were incubated with 2 µg/mL of hTfRMAb-1 and -2 for 60 min at 37 °C. After washing with PBS(+), the spheroids were fixed with 4% paraformaldehyde. Internalized hTfRMAbs and lysosomes in HBMEC/ci18 cells of the hMCS-BBB models were observed using the FV3000 confocal microscope. Co-localization of hTfRMAb-1 and -2 with lysosomes was examined using the Olympus CellSens imaging software, and their co-localization rates were calculated via Pearson’s correlation coefficient analyses.

### Statistical analysis

Student’s *t*-test was conducted using Microsoft Excel (Office 365) to analyze the statistical significance of the difference between two values.

## Results

### Generation of HBMEC/ci18^TfR − KO^ cells

**Fig. 1:**
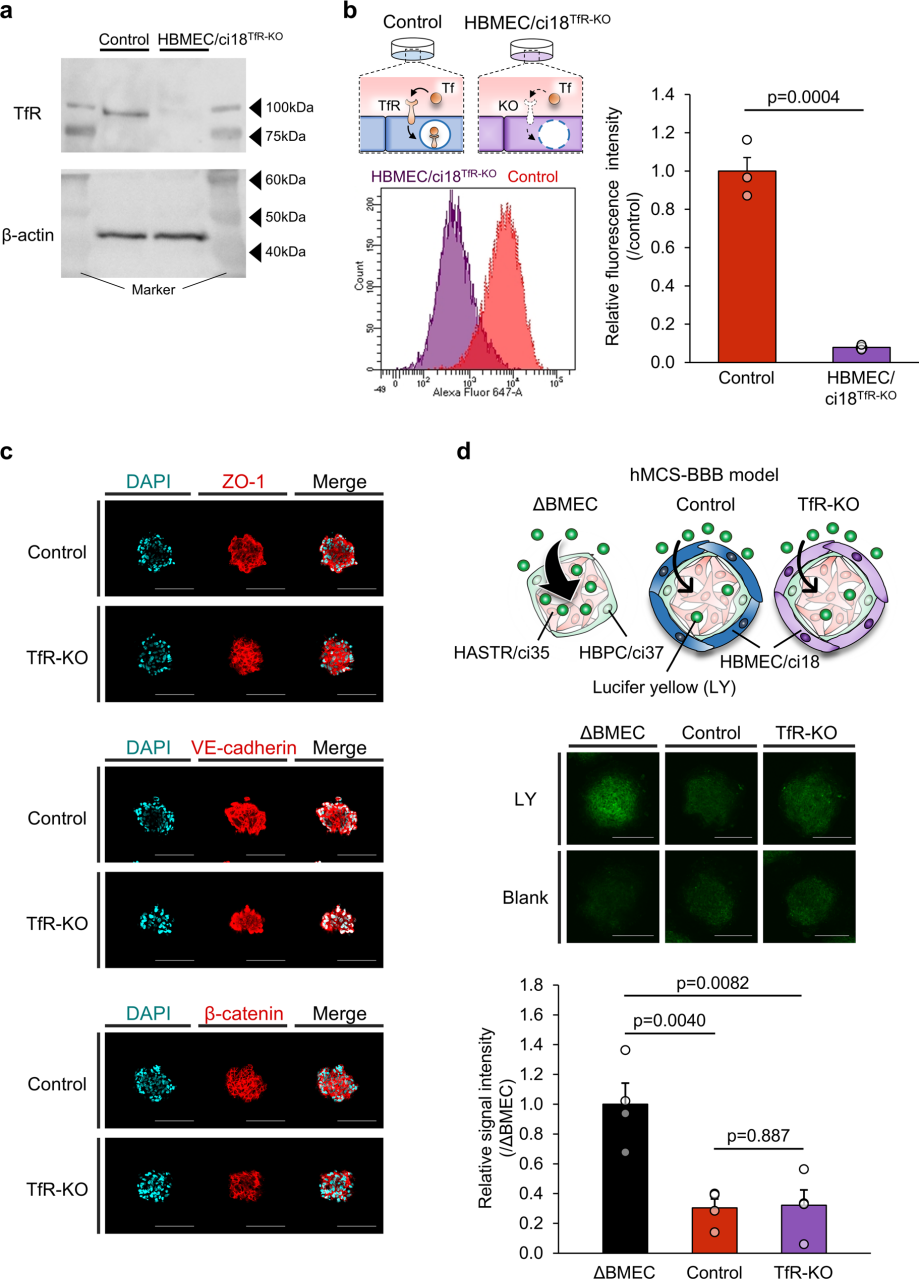
Generation of TfR-KO cell lines and their impact on the hMCS-BBB model barrier functions. (**a**) Protein expression levels of TfR in the whole-cell lysates (0.5 µg/lane) of the control and HBMEC/ci18^TfR − KO^ cells were examined by Western blotting. β-actin was used as a loading control. Representative results of two biologically independent experiments are shown (the uncropped images are shown in Fig. S2). (**b**) Alexa Fluor 647-conjugated Tf (500 nM) was added to the monocultured control and HBMEC/ci18^TfR − KO^ cells and incubated at 37 °C for 20 min. After incubation, the cells were collected, and fluorescence intensity of each cell was measured by flow cytometry. Fluorescence intensity values were determined by subtracting the values of the blank cells (background) from those of the test cells. Relative fluorescence intensities (HBMEC/ci18^TfR − KO^ /control cells, where control cell value = 1) were also calculated and are expressed as the mean ± standard error of the mean (SEM; three biologically independent experiments). Statistical analyses were conducted via Student’s *t*-test. (**c**) Representative ICC images of the control and TfR-KO models for ZO-1, VE-cadherin, and β-catenin from two independent experiments (red). DAPI was used for nuclear counterstaining (blue). The same microscopic parameters were used for fluorescence detection in comparative analyses. Scale bar, 150 μm. (**d**) LY permeability assays of the control and TfR-KO models. Models without HBMEC/ci18 cells (ΔBMEC models) were also prepared for comparison. After 90-min incubation with the HBSS(+) containing LY (50 µM), the spheroids were subjected to microscopic analyses. Fluorescence signal intensity in the central area of each test spheroid was determined by subtracting those of the blank spheroids from those of the test spheroids. Average relative signal intensities (fluorescence intensity of the ΔBMEC model = 1) were calculated using five spheroids, and the results are represented as the mean ± SEM of four biologically independent experiments. Statistical analyses were conducted via Student’s *t*-test. Scale bar, 150 μm

To clarify the role of TfR in hTfRMAb uptake by hMCS-BBB models and to characterize the associated kinetics, it is essential to isolate the fraction specifically attributable to TfR-dependent transcytosis. First, we generated HBMEC/ci18^TfR − KO^ cells using the CRISPR-Cas9 system. After isolating several HBMEC/ci18^TfR − KO^ clones, their TfR protein expression levels were analyzed by Western blotting, revealing that clone 19 did not express TfR (Fig. 1a). TfR-KO of this clone was further validated by a Tf uptake assay, with the uptake level measured at 7.9 ± 0.6%, compared to 100.0 ± 7.0% in the parental cells (Fig. 1b). These results confirmed the loss of functional TfR expression in the tested clone.

Next, we examined whether TfR-KO affected the barrier functions of the hMCS-BBB models. ICC results revealed that the protein expression profiles of ZO-1 (a tight junction protein), as well as VE-cadherin and β-catenin (adherens junction proteins), were comparable between the TfR-KO (i.e., models incorporating HBMEC/ci18^TfR − KO^ cells) and control models (Fig. 1c). LY permeability assays were performed to further assess their paracellular barrier functions. Spheroid models lacking HBMEC/ci18 cells (ΔBMEC models), which were expected to exhibit no barrier functions, were also prepared as negative controls. LY permeability levels in both the control (0.3 ± 0.1-fold) and TfR-KO (0.3 ± 0.1-fold) models were comparably lower than those in the ΔBMEC models (1.0 ± 0.1-fold; Fig. 1d). Collectively, these findings suggest that TfR-KO does not compromise the barrier integrity of the hMCS-BBB models.

### hTfRMAb BBB permeability assays using hMCS-BBB models

**Fig. 2:**
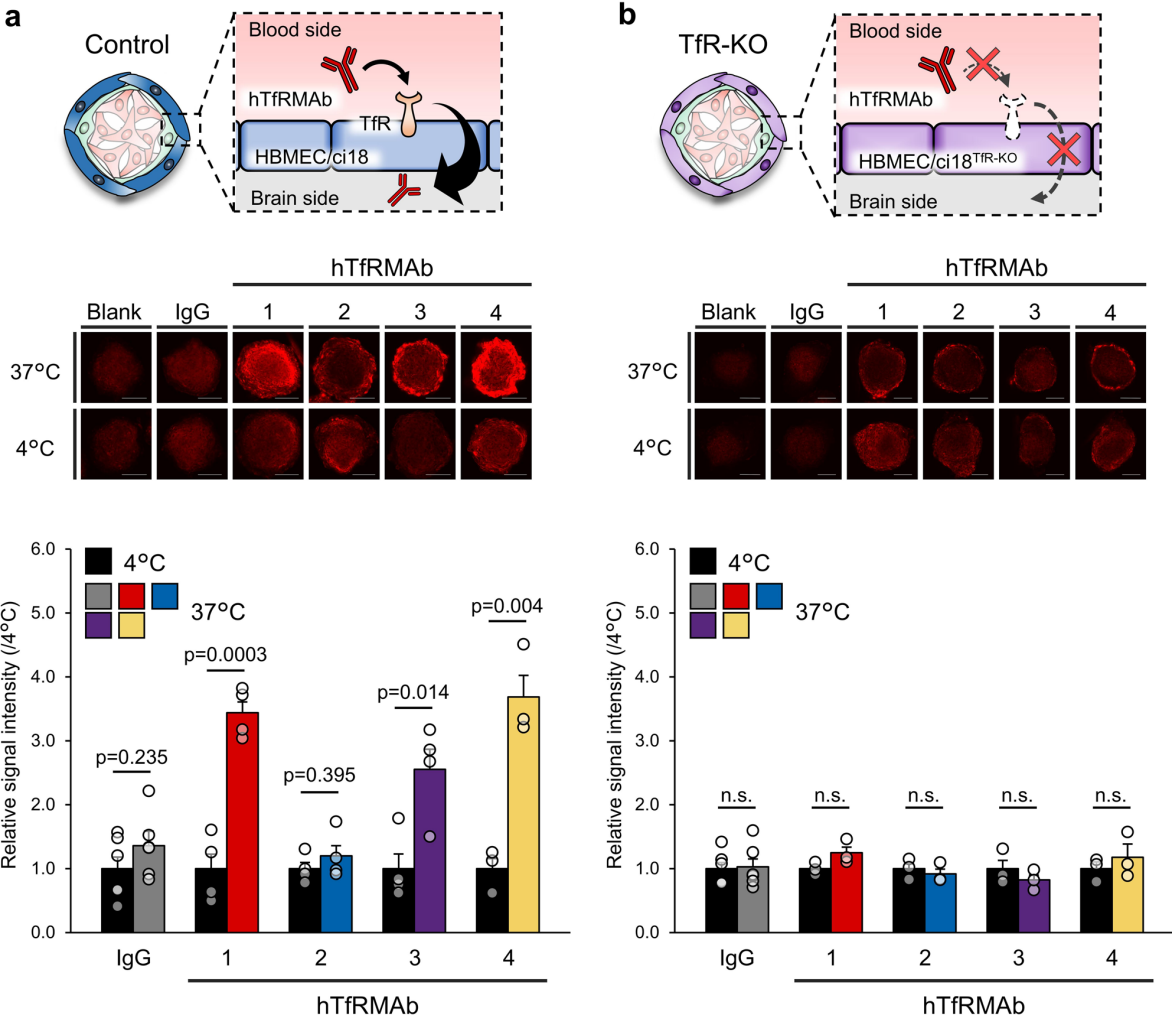
BBB permeability assays for four different hTfRMAbs using the control and TfR-KO models. (**a** and **b**) Permeability levels of HiLyte Fluor 647-labeled hTfRMAbs (hTfRMAb-1, -2, -3, and -4; each 2 µg/mL) and isotype control IgG (2 µg/mL) were examined at 37 °C and 4 °C using the control (**a**) and TfR-KO (**b**) models. After 2-h incubation in a SFM with iron-saturated holo-Tf (25 µM) and each antibody, fluorescence signals in the central area of each test spheroid were detected using confocal microscopy, and the signal values were determined by subtracting those of the corresponding blank spheroids from those of the test spheroids. Relative signal intensities (37 °C/4°C condition, where 4 °C condition value = 1) were calculated and are expressed as the mean ± SEM (*n* = 5 spheroids; at least three biologically independent experiments). Statistical analyses were conducted using Student’s *t*-test. Scale bar, 100 μm

To evaluate the potential of the hMCS-BBB models to assess the BBB permeability of hTfRMAbs, permeability assays were performed using four different hTfRMAbs (hTfRMAb-1, -2, -3, and -4) and an isotype control IgG. As shown in Table 1, all four hTfRMAbs exhibited very high and comparable affinities for hTfR, with K_D_ values ranging from 2.35 × 10^− 11^ M to below the detection limit (< 10^− 12^ M). Despite this, the evaluation results using the hMCS-BBB models revealed that the four hTfRMAbs exhibit distinct BBB permeability profiles. hTfRMAb-1, -3, and -4 possess clear BBB permeability (uptake activities: 3.4 ± 0.2, 2.6 ± 0.3, and 3.7 ± 0.3, respectively) (Fig. 2a; Table 1), whereas the uptake activity of hTfRMAb-2 was comparable to that of the control IgG (1.2 ± 0.2 and 1.4 ± 0.2, respectively).

**Table 1:** Binding properties of hTfRMAbs to hTfR and their BBB permeability

Antibody	K_D_ values for binding to hTfR	Relative in vitro BBB permeability	Km,_TfR_ values of in vitro BBB permeability
	(M)^a^	(4℃ = 1.0 ± SEM)	(µg/mL)
hTfRMAb-1	2.35 × 10^− 11^	3.44 ± 0.17	6.48
hTfRMAb-2	< 1.0 × 10^− 12^	1.20 ± 0.16	-
hTfRMAb-3	< 1.0 × 10^− 12^	2.55 ± 0.32	-
hTfRMAb-4	< 1.0 × 10^− 12^	3.69 ± 0.34	12.02

^*a*^, The association rate constants (K_on_) and dissociation rate constants (K_off_) are shown in Table S4

Similar permeability assays were performed using TfR-KO models to determine the role of TfR in the BBB permeability of the hTfRMAbs. In contrast to the results obtained using wild-type hMCS-BBB models, uptake activity levels of hTfRMAb-1, -3, and -4 in TfR-KO models were as low as those observed for hTfRMAb-2 and control IgG (1.0 ± 0.1-, 1.2 ± 0.1-, 0.9 ± 0.1-, 0.8 ± 0.1-, and 1.2 ± 0.2-fold for control IgG, hTfRMAb-1, -2, -3, and -4, respectively; Fig. 2b). These findings confirmed that the BBB permeability of hTfRMAb-1, -3, and -4 in our models was mediated via the TfR-dependent transcytosis pathway.

Given the differences in BBB permeability among hTfRMAbs, we compared the in vitro results with previously reported in vivo data from monkeys (IVIVC analysis). In the in vivo studies (Supplemental materials and methods, Table S2, and Fig. S1 [28]), the brain concentrations of hTfRMAb-1, -3, and -4 after intravenous administration were significantly higher than that of hTfRMAb-2, indicating that the former three are BBB-permeable, whereas the latter is not. Our in vitro results were in good agreement with these in vivo findings.

Collectively, these findings suggest that the hMCS-BBB models can effectively assess the differences in the TfR-mediated BBB permeability of hTfRMAbs and yield results that closely reflect their in vivo behavior.

### Characterization of the BBB permeability kinetic properties of brain-penetrating hTfRMAbs using hMCS-BBB models

**Fig. 3:**
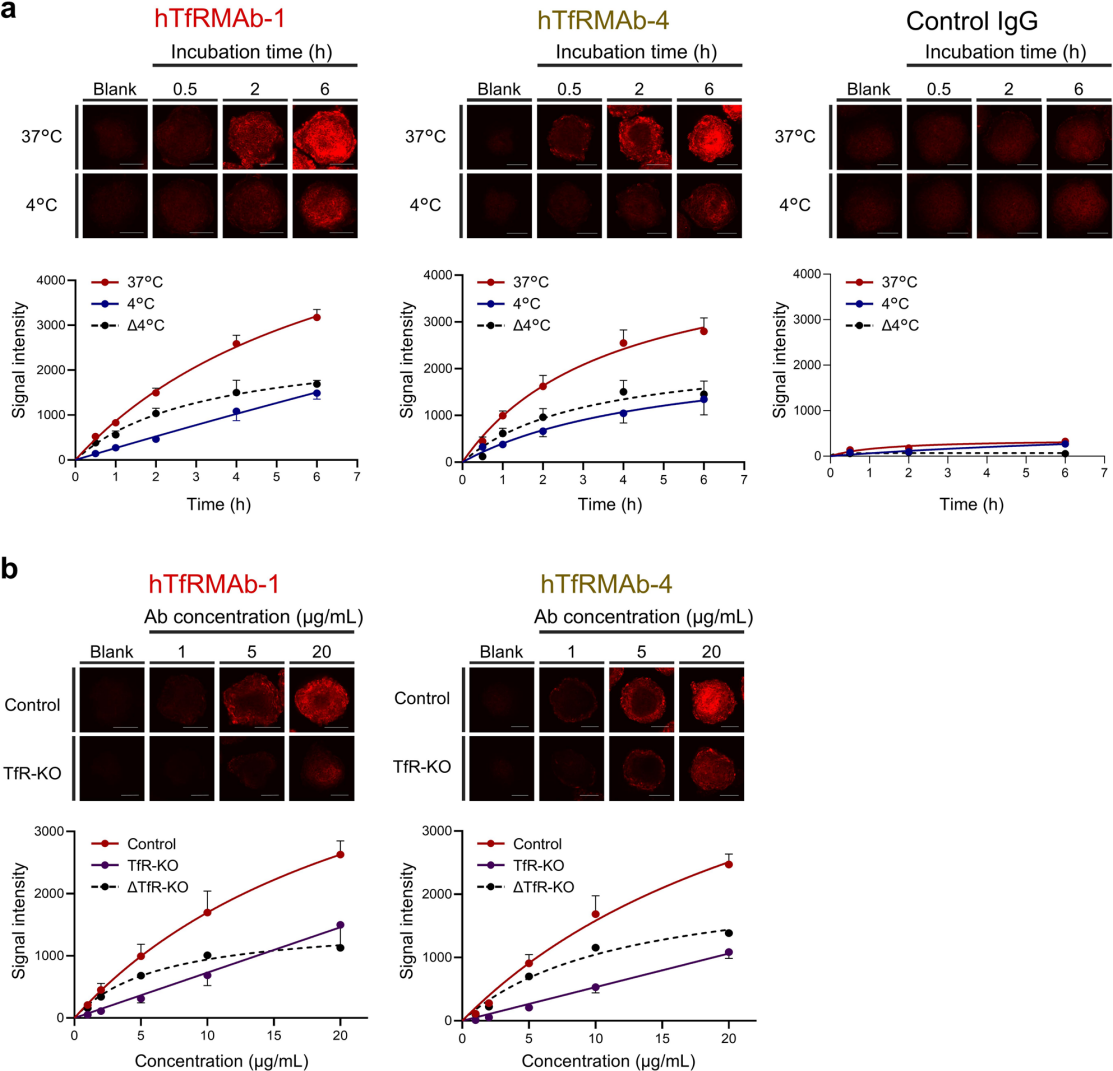
Analysis of the BBB permeability kinetic properties of brain-penetrant hTfRMAbs. (**a**) Time-dependent analysis of hTfRMAb-1, hTfRMAb-4, and control IgG at 0.5, 1, 2, 4, and 6 h. Fluorescence at each time point was detected using confocal microscopy, and fluorescence intensities were determined as described above. The microscopic settings used to acquire all fluorescence images were kept identical throughout the analyses. Their mean ± SEM values (*n* = 5; three biologically independent experiments) were plotted to draw the fitting curves using Prism8 (red line: 37 °C; blue line: 4 °C; black dotted line: Δ37°C = signal intensity at 37 °C – signal intensity at 4 °C). Scale bar, 100 μm. (**b**) Concentration-dependent analysis of hTfRMAb-1 and -4 at 1, 2, 5, 10, and 20 µg/mL. TfR-KO models were prepared in addition to control models to evaluate the contribution of TfR to the BBB permeability of hTfRMAbs. The ΔTfR curve was generated by subtracting the fluorescence intensity of the TfR-KO models at 37 °C from that of the control models at 37 °C at each concentration point (red line: control models; purple line: TfR-KO models; black dotted line: ΔTfR). Scale bar, 100 μm

Generally speaking, single-point analysis does not provide sufficient information for the development of BBB-permeable antibodies. Therefore, we investigated whether the hMCS-BBB models can be used to assess the kinetic properties of hTfRMAb-1 and -4 uptake. Control IgG was used for comparison. Time-dependent uptake profiles (0.5–6 h) of hTfRMAb-1 and -4 were generated by subtracting the fluorescence intensity at 4 °C from that at 37 °C (dotted line, Δ4°C). The linear uptake ranges were up to 2 h for hTfRMAb-1 and 1 h for hTfRMAb-4 (Fig. 3a).

Next, concentration-dependent uptake profiles (1–20 µg/mL) of hTfRMAb-1 and -4 were evaluated under the time conditions determined above. The results showed that both hTfRMAb-1 and -4 were taken up in a saturable manner. To further characterize the TfR-dependent hTfRMAb uptake pathway, a ΔTfR curve (black dotted line) was plotted by subtracting the uptake levels in the TfR-KO spheroids from those in the control spheroids. Michaelis–Menten curve fitting was applied to the ΔTfR data, revealing distinct BBB permeability properties, with *Km*,_*TfR*_ values of 6.5 and 12.0 µg/mL for hTfRMAb-1 and -4, respectively (Fig. 3b; Table 1).

These results show that the hMCS-BBB models can distinguish the distinct kinetic properties of hTfRMAb-1 and -4.

### hTfRMAb uptake assays using monocultured HBMEC/ci18 cells

**Fig. 4:**
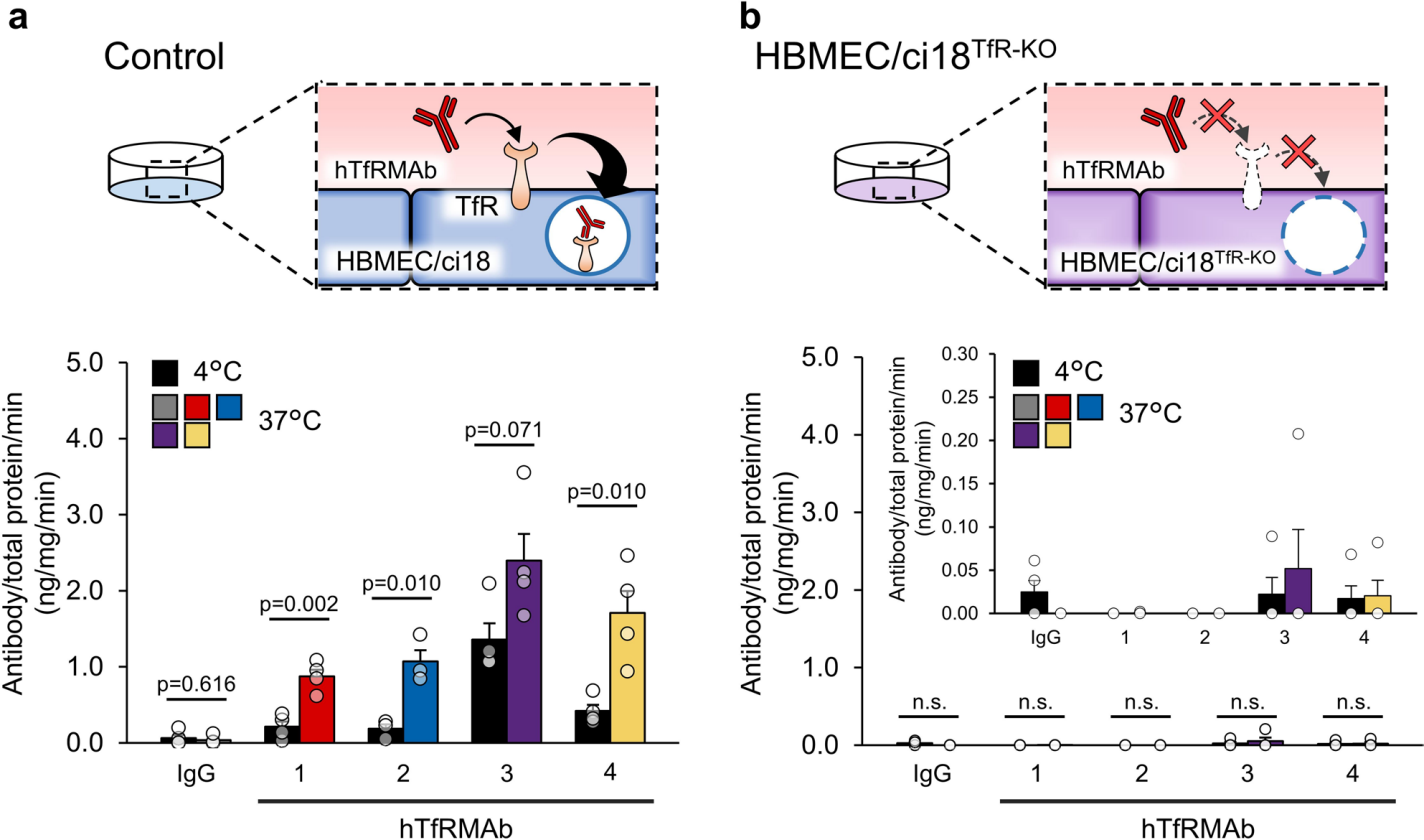
hTfRMAb uptake assays using monocultured HBMEC/ci18 cells. (**a** and **b**) Control (**a**) and HBMEC/ci18^TfR − KO^ (**b**) cells were seeded at 3.0 × 10^4^ cells/well in 12-well plates and cultured for 24 h at 37 °C. After the addition of each fluorescently labeled hTfRMAb or control IgG at 1.0 µg/mL, the cells were incubated at either 4 °C or 37 °C for 20 min. After careful washing with PBS(–), the cell lysates were prepared, and their fluorescence intensity was measured. hTfRMAb uptake activity (antibody level [ng/mL]/total protein concentration [mg/mL]/min) was calculated and expressed as the mean ± SEM (at least three biologically independent experiments). Statistical analyses were conducted using Student’s *t*-test

The above-mentioned results highlight the applicability of hMCS-BBB models for hTfRMAb development; however, it is possible that HBMEC/ci18 cells alone could yield comparable results. To examine this, we performed uptake assays using monocultured HBMEC/ci18 cells. All tested hTfRMAbs, including non-BBB-permeable hTfRMAb-2, were effectively taken up by HBMEC/ci18 cells. Uptake levels of hTfRMAb-1, -2, -3, and -4 (0.9 ± 0.1, 1.1 ± 0.1, 2.4 ± 0.3, and 1.7 ± 0.3 ng/total protein mg/min, respectively) were significantly higher than that of the control IgG (0.03 ± 0.02) at 37 °C (Fig. 4a).

To verify the involvement of TfR in the uptake of all hTfRMAbs by HBMEC/ci18 cells, similar uptake assays were conducted using HBMEC/ci18^TfR − KO^ cells. Indeed, minimal or no energy-dependent cellular uptake of the tested hTfRMAbs was observed (Fig. 4b), suggesting that TfR primarily mediates hTfRMAb uptake by HBMEC/ci18 cells.

Overall, these findings suggest that differences in BBB permeability among hTfRMAbs cannot always be determined by uptake assays using simple HBMEC/ci18 monoculture systems, as these assays primarily assess the initial endocytosis process. In contrast, the hMCS-BBB models successfully distinguished these permeability differences in an in vivo-corelated manner, highlighting their practical significance for novel hTfRMAb development.

### Intracellular localization analysis of hTfRMAb using hMCS-BBB models

BBB permeability differences among the tested hTfRMAbs were possibly due to variations in any of the three RMT pathway steps: Endocytosis, intracellular transport, and exocytosis. However, based on the results described above, differences in susceptibility to endocytosis alone possibly do not explain the variations in the BBB permeability of hTfRMAbs. It has been reported that, compared to BBB-permeable hTfRMAbs, non-BBB-permeable hTfRMAbs are more likely to undergo lysosomal degradation following endocytosis [29–31]. Since a similar mechanism may underlie the observations of this study, we performed co-localization analysis of hTfRMAb-1 and -2 with lysosomes in HBMEC/ci18 cells using hMCS-BBB models.

**Fig. 5:**
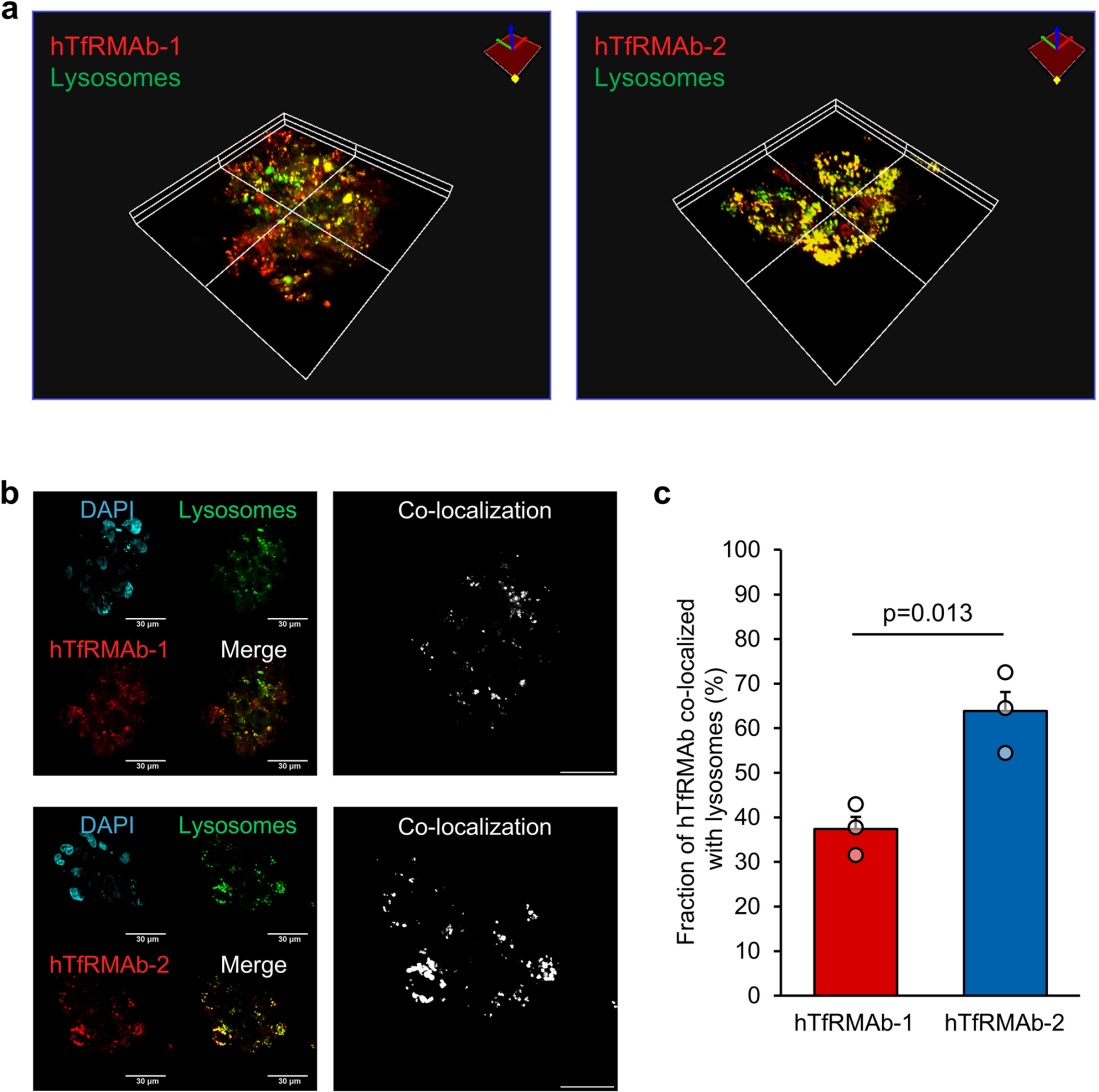
Co-localization analysis of hTfRMAbs with lysosomes using hMCS-BBB models. In the HBMEC/ci18 cells of the hMCS-BBB model, co-localization analysis was performed between HiLight Fluor 647-labeled hTfRMAb-1 or -2 (red) with lysosomes (stained with LysoTracker; green) (obtained from three biologically independent experiments). (**a**) Representative three-dimensional images showing the co-localization of each hTfRMAb with lysosomes. (**b**) Representative images showing the co-localization of each hTfRMAb with lysosomes at a depth of 4.0 μm from the top of the spheroids. Nuclei were labeled with DAPI (blue). Images in the right panel show the co-localized vesicles in white. Scale bar, 30 μm. (**c**) The hTfRMAb-1 or -2 fractions co-localized with lysosomes were quantified. The graph shows the lysosomal co-localization rate of hTfRMAb-1 or -2, and the results are represented as the mean ± SEM (at least five images from three biologically independent experiments). Co-localization analysis was performed using the Olympus CellSens imaging software. Statistical analyses were conducted using Student’s *t*-test

As shown in the microscopic images, compared to those of non-BBB-permeable hTfRMAb-2, intracellular fluorescent signals of BBB-permeable hTfRMAb-1 were largely separated from those of the lysosomes (stained by LysoTracker; Fig. 5a and b). Quantitative analysis confirmed this observation, revealing that the lysosomal co-localization rate of hTfRMAb-1 was significantly lower (37.4 ± 2.7%; Pearson’s correlation coefficient (R) = 0.32 ± 0.01) than that of hTfRMAb-2 (63.8 ± 4.3%; *R* = 0.45 ± 0.01; Fig. 5c). Therefore, hTfRMAb-1 exhibits a relatively low trafficking rate through the lysosomal degradation pathway, partly explaining its enhanced ability to penetrate the BBB.

## Discussion

Early prediction of human BBB permeability of brain-penetrant antibodies is crucial for their successful development. Although hMCS-BBB models exhibit the potential to predict the BBB permeability of antibodies [19], their evaluative capacity and practical applications remain insufficiently characterized. Therefore, we extended our previous work to assess their applicability in the development of brain-penetrant antibodies. The main findings of this study are as follows: hMCS-BBB models (1) demonstrate the potential to extrapolate hTfRMAb BBB permeability data to in vivo settings; (2) facilitate the kinetic characterization of hTfRMAb BBB permeability; and (3) provide insights into the intracellular transport mechanisms underlying the varying BBB permeability of hTfRMAbs.

The first finding will have a substantial impact on related studies. For practical applications, BBB models, including hMCS-BBB models, must generate results that can be appropriately translated into in vivo outcomes, and those lacking in vivo extrapolation capability have little practical value, regardless of their structural similarity to in vivo conditions. Although many MPS-based BBB models have been developed [15–17], few studies have directly assessed their in vivo extrapolation capability — even for hTfRMAbs, despite being among the most actively investigated brain-targeting antibodies. Limited examples include the studies by Burgio et al. [32] and Chim et al. [33], who investigated the correlations between in vitro BBB permeability data of selected modalities and in vivo results obtained from mice. However, no previous studies have demonstrated correlations between in vitro BBB permeability data for hTfRMAbs and in vivo results obtained from primates. Considering the close genetic similarity between primates and humans, IVIVCs established in this study encourage the use of hMCS-BBB models for reliably predicting human BBB permeability of hTfRMAbs. Nevertheless, the sample size in this study is limited; therefore, further investigations using a broader range of BBB-penetrant hTfRMAbs are necessary to strengthen the in vivo extrapolation capability of the hMCS-BBB models.

The second point — that hMCS-BBB models can yield kinetic parameters for BBB penetration of hTfRMAbs — offers essential input for the construction of physiologically based pharmacokinetic models of the central nervous system (CNS-PBPK). Although several CNS-PBPK models aimed at predicting brain concentrations of hTfRMAbs have been described [34–36], parameters derived from BBB models (including, but not limited to, ours) have not yet been incorporated. While caution is warranted, these parameters may serve as initial parameter estimates for CNS-PBPK modeling. Incorporating BBB model-derived parameters into CNS-PBPK modeling is considered a necessary step toward improving the accuracy of brain concentration predictions, with hMCS-BBB models expected to play a significant role.

The kinetic parameter data of hTfRMAbs provide valuable insights beyond merely assessing their BBB permeability, contributing to their overall development. Considering the blood concentration profiles, *Km*,_*TfR*_ values serve as useful parameters for estimating the brain influx of hTfRMAbs. For example, when simulating a blood concentration range of 5–15 µg/mL for hTfRMAb-1 after intravenous administration, the predicted BBB penetration would fall within the saturated phase, as determined by its *Km*,_*TfR*_ value (6.5 µg/mL; Fig. 3b; Table 1). In contrast, in a similar simulation for hTfRMAb-4, its BBB penetration level would increase as blood concentration rises, reflecting its *Km*,_*TfR*_ value (12.0 µg/mL; Fig. 3b; Table 1). Therefore, hTfRMAb-4 could be a promising brain-penetrating antibody in the presence of sufficiently high blood concentrations. Notably, blood concentrations used in these simulations are clinically relevant, as they are comparable to the reported *Cmax* (11.3 ± 6.8 µg/mL) following a single 2.0 mg/kg intravenous dose of pabinafusp alfa in humans [37]. Collectively, these simulations using kinetic parameters can aid in decision making during early hTfRMAb development stages.

In addition to the above-mentioned points, our results provide important insights into the mechanisms underlying the BBB permeability of hTfRMAbs (the third point). First, the extremely high affinities for hTfR observed with the bivalent format of all four hTfRMAbs do not appear to align with the proposed fine-tuning TfR affinity strategy [38–40]. Based on PBPK model simulations for TfR-targeting antibodies in non-human primates, the optimal affinities have been proposed to fall within the range of 100 − 10,000 nM [36] or 800-3,000 nM [41]. Although these prediction primarily focus on monovalent formats, the estimated optimal values are remarkably higher (more than 1,000-fold) than those of the hTfRMAbs tested in this study. Our findings suggest that binding affinity serves as only one of the key factors determining BBB penetration properties, while others may play more significant roles in certain cases. For example, the overall structure of the TfR-hTfRMAb complex on the cell surface, as well as within endosomal compartments, may act as such factors. It can also be speculated that transcytosis-associated accessory proteins may vary depending on the complex structure.

Furthermore, our co-localization assays reveal that, despite sharing a common bivalent mouse IgG format and differing only in their Fab domains, hTfRMAb-1 and hTfRMAb-2 exhibited distinct lysosomal colocalization rates (37.4% and 63.8%, Fig. 5c). Given that endogenous IgG exhibits a lysosomal colocalization rate of approximately 20% [42], these findings suggest that Fab-mediated interactions, rather than the IgG backbone itself, play a key role in determining lysosomal trafficking fate. The observation is consistent with previous reports indicating that non-BBB-permeable hTfRMAbs are more likely to undergo lysosomal degradation [29–31]. Rab family GTPases are possibly involved in the underlying pathways; however, the precise mechanisms remain ambiguous. Villaseñor et al. [43] have reported that a TfR-targeting brain shuttle (monovalent Fab) co-localizes with Rab17 to form sorting tubules, which facilitate transcytosis across the BBB. Furthermore, Haqqani et al. [44] have reported that brain-permeable hTfRMAbs are more commonly associated with Rab5a than Rab7a, whereas non-permeable hTfRMAbs co-localize with Rab7a or lysosomes. These reports suggest the hypothesis that specific Rab association patterns, reflecting the distinct steps in intracellular trafficking, could determine the transcytotic efficiency of hTfRMAbs.

In any event, our findings provide a unique example that encourages consideration of the behavior of the TfR-antibody complex throughout the transcytosis process, integrating binding affinity, epitope recognition, and context-dependent structural dynamics for successful BBB penetration.

Finally, we would like to highlight another key aspect of this study: the differences in the in vivo BBB permeability of the four hTfRMAbs were successfully recapitulated using hMCS-BBB models, but not with simple mono-cultured HBMEC/ci18 cells. Although the specific factors responsible for the distinct predictive performance of hMCS-BBB models remain unknown, their structural and cellular complexities are possibly involved. The microenvironment of hMCS-BBB models, which allow direct cell–cell contact in a three-dimensional structure, possibly promote BBB maturation. We previously demonstrated the polarized expression of P-glycoprotein and elevated levels of TfR and some barrier-related genes in hMCS-BBB models compared to those in transwell-based models. Such in vivo-like microenvironments facilitate the formation of polarized intracellular trafficking pathways [17, 45], which possibly affects the efficiency of hTfRMAb transcytosis across the BBB. Although the underlying mechanisms warrant further investigation, our findings provide compelling evidence of the advantages of MPS-based BBB models for the development of brain-penetrant antibodies.

## Conclusions

In summary, hMCS-BBB models effectively characterized the BBB permeability of hTfRMAbs, distinguishing permeable from non-permeable antibodies despite their similarly high target affinities, and providing insights into their kinetic and mechanistic differences. Notably, the observed differences in BBB permeability among tested hTfRMAbs are closely correlated with their in vivo permeability profiles, which are difficult to obtain using simple HBMEC/ci18 monoculture uptake assays. Therefore, hMCS-BBB models are promising tools for the development of BBB-permeable antibodies with reliable in vivo extrapolation capacity. To strengthen the reliability of the current findings and broaden the applicability of the BBB models to other macromolecular modalities, further investigation is warranted.

## Supplementary Information

Below is the link to the electronic supplementary material.

Supplementary Material 1

## Data Availability

The data that support the findings of this study are available from the corresponding author upon reasonable request.

## Abbreviations

BBB: Blood–brain barrier
BMEC: Brain microvascular endothelial cells
CNS: Central nervous system
CNS-PBPK: Central nervous system physiologically based pharmacokinetic
CRISPR-Cas9: Clustered regularly interspaced short palindromic repeats-associated protein 9
DAPI: 4’,6-diamidino-2-phenylindole
DDS: Drug delivery system
EC_50_: Half maximal effective concentration
ELISA: Enzyme-linked immunosorbent assay
Fab: Fragment antigen binding
HASTR/ci35: Human astrocytes/conditionally immortalized clone 35
HBMEC/ci18: Human brain microvascular endothelial cells/conditionally immortalized clone 18
HBPC/ci37: Human brain pericytes/conditionally immortalized clone 37
HBSS: Hanks’ balanced salt solution
hMCS-BBB: Human multi-cellular spheroidal blood-brain barrier
HRP: Horseradish peroxidase
hTfRMAb: Anti-human transferrin receptor monoclonal antibody
ICC: Immunocytochemistry
IVIVC: In vitro–in vivo correlation
K_D_: Dissociation constant
Km: Michaelis constant
KO: Knockout
LY: Lucifer yellow
MPS: Microphysiological system
PBS: Phosphate-buffered saline
RMT: Receptor-mediated transcytosis
SEM: Standard error of the mean
SFM: Serum-free medium
sgRNA: Single guide RNA
Tf: Transferrin
TfR: Transferrin receptor
TMB: 3,3’,5,5’-tetramethylbenzidine
ZO-1: Zonula occludens-1

## References

[1] Abbott NJ. Dynamics of CNS barriers: evolution, differentiation, and modulation. Cell Mol Neurobiol. 2005;25(1):5–23. 15962506 10.1007/s10571-004-1374-y PMC11529509

[2] Wu D, et al. The blood-brain barrier: structure, regulation, and drug delivery. Signal Transduct Target Ther. 2023;8(1):217. 37231000 10.1038/s41392-023-01481-w PMC10212980

[3] Liebner S, et al. Functional morphology of the blood-brain barrier in health and disease. Acta Neuropathol. 2018;135(3):311–36. 29411111 10.1007/s00401-018-1815-1 PMC6781630

[4] Pardridge WM. Receptor-mediated drug delivery of bispecific therapeutic antibodies through the blood-brain barrier. Front Drug Deliv. 2023;3:1227816. 37583474 10.3389/fddev.2023.1227816 PMC10426772

[5] Kay AD, et al. CSF and serum concentrations of albumin and IgG in alzheimer’s disease. Neurobiol Aging. 1987;8(1):21–5. 3561662 10.1016/0197-4580(87)90053-4

[6] Friden PM, et al. Anti-transferrin receptor antibody and antibody-drug conjugates cross the blood-brain barrier. Proc Natl Acad Sci USA. 1991;88(11):4771–5. 2052557 10.1073/pnas.88.11.4771 PMC51748

[7] Baghirov H. Mechanisms of receptor-mediated transcytosis at the blood-brain barrier. J Control Release. 2025;381:113595. 40056994 10.1016/j.jconrel.2025.113595

[8] Sonoda H, et al. A blood-brain-barrier-penetrating anti-human transferrin receptor antibody fusion protein for neuronopathic mucopolysaccharidosis II. Mol Ther. 2018;26(5):1366–74. 29606503 10.1016/j.ymthe.2018.02.032 PMC5993955

[9] Giugliani R, et al. Iduronate-2-sulfatase fused with anti-hTfR antibody, pabinafusp alfa, for MPS-II: A phase 2 trial in Brazil. Mol Ther. 2021;29(7):2378–86. 33781915 10.1016/j.ymthe.2021.03.019 PMC8261166

[10] Okuyama T, et al. A phase 2/3 trial of pabinafusp alfa, IDS fused with anti-human transferrin receptor antibody, targeting neurodegeneration in MPS-II. Mol Ther. 2021;29(2):671–9. 33038326 10.1016/j.ymthe.2020.09.039 PMC7854283

[11] Grimm HP, et al. Delivery of the Brainshuttle™ amyloid-beta antibody fusion trontinemab to non-human primate brain and projected efficacious dose regimens in humans. MAbs. 2023;15(1):2261509. 37823690 10.1080/19420862.2023.2261509 PMC10572082

[12] Zhang W, et al. Differential expression of receptors mediating receptor-mediated transcytosis (RMT) in brain microvessels, brain parenchyma and peripheral tissues of the mouse and the human. Fluids Barriers CNS. 2020;17(1):47. 32698806 10.1186/s12987-020-00209-0 PMC7376922

[13] Ross SR, et al. Mouse transferrin receptor 1 is the cell entry receptor for mouse mammary tumor virus. Proc Natl Acad Sci USA. 2002;99(19):12386–90. 12218182 10.1073/pnas.192360099 PMC129454

[14] Haqqani SA, et al. Receptor-mediated transcytosis for brain delivery of therapeutics: receptor classes and criteria. Front Drug Deliv. 2024;4:1360302. 40836978 10.3389/fddev.2024.1360302 PMC12363266

[15] Kimura H, et al. Microphysiological systems: exploring organoids and organ-on-a-chip technologies in drug development - focus on pharmacokinetics related organs. Drug Metab Pharmacokinet. 2025;60:101046. 39847980 10.1016/j.dmpk.2024.101046

[16] Tran M, et al. Human mini-blood-brain barrier models for biomedical neuroscience research: a review. Biomater Res. 2022;26(1):82. 36527159 10.1186/s40824-022-00332-z PMC9756735

[17] Pérez-López A, et al. An overview of in vitro 3D models of the blood-brain barrier as a tool to predict the in vivo permeability of nanomedicines. Adv Drug Deliv Rev. 2023;196:114816. 37003488 10.1016/j.addr.2023.114816

[18] Kitamura K, et al. Development, characterization and potential applications of a multicellular spheroidal human blood-brain barrier model integrating three conditionally immortalized cell lines. Biol Pharm Bull. 2021;44(7):984–91. 33896887 10.1248/bpb.b21-00218

[19] Kitamura K, et al. Human immortalized cell-based blood-brain barrier spheroid models offer an evaluation tool for the brain penetration properties of macromolecules. Mol Pharm. 2022;19(8):2754–64. 35766901 10.1021/acs.molpharmaceut.2c00120

[20] Ito R, et al. A human immortalized cell-based blood-brain barrier triculture model: development and characterization as a promising tool for drug-brain permeability studies. Mol Pharm. 2019;16(11):4461–71. 31573814 10.1021/acs.molpharmaceut.9b00519

[21] Umehara K, et al. A new conditionally immortalized human fetal brain pericyte cell line: establishment and functional characterization as a promising tool for human brain pericyte studies. Mol Neurobiol. 2018;55(7):5993–6006. 29128907 10.1007/s12035-017-0815-9

[22] Furihata T, et al. Establishment and characterization of a new conditionally immortalized human astrocyte cell line. J Neurochem. 2016;136(1):92–105. 26365151 10.1111/jnc.13358

[23] Kitamura K, et al. Differentiated HASTR/ci35 cells: A promising in vitro human astrocyte model for facilitating CNS drug development studies. J Pharmacol Sci. 2018;137(4):350–8. 30150146 10.1016/j.jphs.2018.06.013

[24] Isogai R, et al. Generation of a human conditionally immortalized cell-based multicellular spheroidal blood-brain barrier model for permeability evaluation of macromolecules. Bio Protoc. 2022;12(15):e4465. 36082368 10.21769/BioProtoc.4465 PMC9411012

[25] Simonneau C, et al. Investigating receptor-mediated antibody transcytosis using blood-brain barrier organoid arrays. Fluids Barriers CNS. 2021;18:43. 34544422 10.1186/s12987-021-00276-x PMC8454074

[26] Kamiichi A, et al. Establishment of a new conditionally immortalized cell line from human brain microvascular endothelial cells: a promising tool for human blood-brain barrier studies. Brain Res. 2012;1488:113–22. 23041702 10.1016/j.brainres.2012.09.042

[27] Ohki S, et al. Functional assessment of immortalized human brain microvascular endothelial cells with different passage numbers: A case study for a prospective proposal on variability management of in vitro blood-brain barrier models. Drug Metab Pharmacokinet. 2025;62:101058. 40184994 10.1016/j.dmpk.2025.101058

[28] Fukatsu T, et al. Evaluation of brain delivery of blood-brain barrier-penetrable anti-human transferrin receptor monoclonal antibodies in monkeys. Exp Anim. 2025;74(Suppl):102. (Meeting abstract).

[29] Choi ES, Shusta EV. Strategies to identify, engineer, and validate antibodies targeting blood-brain barrier receptor-mediated transcytosis systems for CNS drug delivery. Expert Opin Drug Deliv. 2023;20(12):1789–800. 38007619 10.1080/17425247.2023.2286371 PMC10842915

[30] Bien-Ly N, et al. Transferrin receptor (TfR) trafficking determines brain uptake of TfR antibody affinity variants. J Exp Med. 2014;211(2):233–44. 24470444 10.1084/jem.20131660 PMC3920563

[31] Niewoehner J, et al. Increased brain penetration and potency of a therapeutic antibody using a monovalent molecular shuttle. Neuron. 2014;81(1):49–60. 24411731 10.1016/j.neuron.2013.10.061

[32] Burgio F, et al. A perfused in vitro human iPSC-derived blood-brain barrier faithfully mimics transferrin receptor-mediated transcytosis of therapeutic antibodies. Cell Mol Neurobiol. 2023;43(8):4173–87. 37698826 10.1007/s10571-023-01404-x PMC10661771

[33] Chim SM, et al. A human brain-chip for modeling brain pathologies and screening blood-brain barrier crossing therapeutic strategies. Pharmaceutics. 2024;16(10):1314. 39458643 10.3390/pharmaceutics16101314 PMC11510380

[34] Pardridge WM, Chou T. Mathematical models of blood-brain barrier transport of monoclonal antibodies targeting the transferrin receptor and the insulin receptor. Pharmaceuticals. 2021;14(6):535. 34205013 10.3390/ph14060535 PMC8226686

[35] Pardridge WM. Kinetics of blood-brain barrier transport of monoclonal antibodies targeting the insulin receptor and the transferrin receptor. Pharmaceuticals. 2021;15(1):3. 35056060 10.3390/ph15010003 PMC8778919

[36] Sato S, et al. Advanced translational PBPK model for transferrin receptor-mediated drug delivery to the brain. J Control Release. 2023;357:379–93. 37031741 10.1016/j.jconrel.2023.04.012

[37] Okuyama T, et al. Iduronate-2-sulfatase with anti-human transferrin receptor antibody for neuropathic mucopolysaccharidosis II: A phase 1/2 trial. Mol Ther. 2019;27:456–64. 30595526 10.1016/j.ymthe.2018.12.005 PMC6391590

[38] Yu YJ, et al. Boosting brain uptake of a therapeutic antibody by reducing its affinity for a transcytosis target. Sci Transl Med. 2011;3(84):84ra44. 21613623 10.1126/scitranslmed.3002230

[39] Couch JA, et al. Addressing safety liabilities of TfR bispecific antibodies that cross the blood-brain barrier. Sci Transl Med. 2013;5(183):183ra57. 23636093 10.1126/scitranslmed.3005338

[40] Faresjö R, et al. Single domain antibody-scFv conjugate targeting amyloid β and TfR penetrates the blood-brain barrier and interacts with amyloid β. MAbs. 2024;16(1):2410968. 39358860 10.1080/19420862.2024.2410968 PMC11451328

[41] Muliaditan M, et al. Translational minimal physiologically based Pharmacokinetic model for transferrin receptor-mediated brain delivery of antibodies. MAbs. 2025;17(1):2515414. 40568753 10.1080/19420862.2025.2515414 PMC12203839

[42] Villaseñor R, et al. Trafficking of endogenous Immunoglobulons by endothelial cells at the blood-brain barrier. Sci Rep. 2016;6:25658. 27149947 10.1038/srep25658 PMC4858719

[43] Villaseñor R, et al. Sorting tubules regulate blood-brain barrier transcytosis. Cell Rep. 2017;21(11):3256–70. 29241551 10.1016/j.celrep.2017.11.055

[44] Haqqani AS, et al. Intracellular sorting and transcytosis of the rat transferrin receptor antibody OX26 across the blood-brain barrier in vitro is dependent on its binding affinity. J Neurochem. 2018;146(6):735–52. 29877588 10.1111/jnc.14482 PMC6175443

[45] Paranjape AN, et al. A multicellular brain spheroid model for studying the mechanisms and bioeffects of ultrasound-enhanced drug penetration beyond the blood-brain barrier. Sci Rep. 2024;14(1):1909. 10.1038/s41598-023-50203-3 PMC10803331 38253669

