# Supplementary material for: Translational applicability of human blood-brain barrier spheroid models for the development of brain-penetrant therapeutic antibodies
Source: Fluids Barriers CNS. 2026 Jan 24;23:30. doi: 10.1186/s12987-025-00752-8 (PMC12911383; doi:10.1186/s12987-025-00752-8)
Supplement: Supplementary file 1 — Supplementary Material 1 [file 12987_2025_752_MOESM1_ESM.docx]

Translational applicability of human blood-brain barrier spheroid models for the development of brain-penetrant therapeutic antibodies

Seiya Ohki ^1^, Tomoki Fukatsu ^2^, Hideto Morimoto ^2^, Masafumi Kinoshita ^2^, Atsushi Imakiire ^2^, Ryuji Yamamoto ^2^, Hanae Morio ^1^, Hiroyuki Sonoda ^2^, Tomomi Furihata ^1,*^

^1^ Laboratory of Advanced Drug Development Sciences, School of Pharmacy, Tokyo University of Pharmacy and Life Sciences, Tokyo 192-0392, Japan

^2^ JCR Pharmaceuticals, Research Division, Hyogo 659-0021, Japan

*Corresponding author

Tomomi Furihata

Contents

1. Supplementary materials: Supplemental materials and methods
2. Supplementary materials: Tables S1, S2, S3 and S4
3. Supplementary materials: Figures S1 and S2 and their legends

1. Supplementary materials: Supplemental materials and methods

**Animals**

All animal experiments were performed with the approval (approval No. IACUC250-090 and IACUC250-102) of the Institutional Animal Care and Use Committees of JCR Pharmaceuticals (Hyogo, Japan) and Shin Nippon Biomedical Laboratories (Kagoshima, Japan). Monkey experiments were performed in Shin Nippon Biomedical Laboratories.

**Administration of hTfRMAbs to monkeys**

To investigate the pharmacokinetics in monkeys, each hTfRMAb or its vehicle (saline) was administered intravenously (IV) to male cynomolgus monkeys (2–3 years of age) at a dose of 5 mg/kg body weight, as described in the previous study [1, (this is also cited as Ref. #8 in the main text)]. Briefly, 8 hours after dosing, the monkeys were subjected to whole-body perfusion with physiological saline, and their brains were resected. The brain samples were used for both hTfRMAb concentration determination and immunohistochemical analysis.

**Determination of hTfRMAb concentrations in the brain cortex and cerebellum**

Cortex and cerebellum regions of each resected brain was homogenized in a buffer containing a protease inhibitor cocktail using a bead-beater–type homogenizer, and then centrifuged to obtain the soluble fraction of the tissue lysate. The hTfRMAb concentration in each brain sample was quantified using a validated in-house electrochemiluminescent assay system as previously described [1].

**Immunohistochemical analysis of hTfRMAb in the cerebellum**

For immunohistochemical analysis, the 4-µm-thick frozen sections were prepared from the cerebellum embedded in OCT compound. The sections were fixed with 4% paraformaldehyde, immersed in 0.3% H_2_O_2_/methanol solution to quench endogenous peroxidase activity, blocked with 0.05% Tween 20/skim milk for 2 hours, and incubated with HRP-labeled, monkey-adsorbed human IgG antibody (Bethyl) for 2 hours, followed by incubation with the amplification reagent of the CSA II biotin-free tyramide signal amplification system (Dako) for 30 min. Subsequently, the specimens were incubated with an HRP-conjugated anti-fluorescein antibody for 15 min and developed with 3,3’-diaminobenzidine. Counterstaining was performed with hematoxylin.

1. Sonoda H, et al. A blood-brain-barrier-penetrating anti-human transferrin receptor antibody fusion protein for neuronopathic mucopolysaccharidosis II. Mol Ther. 2018;26(5):1366-1374.

2. Supplementary materials: Tables

**Table S1. List of antibodies**

| **Antigen** | **Antibody type** | **Application** | **Dilution** | **Manufacturer** | **Catalog number** |
| --- | --- | --- | --- | --- | --- |
| β-actin | Anti-β-actin mouse  monoclonal antibody | WB | ×5,000 | ProteinTech | 60008-1-Ig |
| TfR | Anti-hTfR mouse  monoclonal antibody | WB | ×625 | -* | -* |
| Mouse IgG | Anti-mouse IgG antibody  (HRP-conjugate) | WB | ×10,000 | Promega | W4021 |
| ZO-1 | Anti-ZO-1 rabbit  polyclonal antibody | ICC | ×200 | Cell Signaling | 5406S |
| VE-cadherin | Anti-VE-cadherin goat polyclonal antibody | ICC | ×200 | Santa Cruz Biotechnology | sc-6458 |
| β-catenin | Anti-β-catenin rabbit  monoclonal antibody | ICC | ×200 | Cell Signaling | 8480S |
| Rabbit IgG | Anti-rabbit IgG antibody  (CF647-conjugate) | ICC | ×200 | Biotium | 20047 |
| Goat IgG | Anti-goat IgG antibody  (CF647-conjugate) | ICC | ×200 | Biotium | 20048 |

*Anti-TfR antibodies for WB were provided by JCR Pharmaceuticals.

Western blotting: WB, Immunocytochemistry: ICC

**Table S2. Brain concentrations of hTfRMAbs in cynomolgus monkeys**

| **Antibody** | **Cerebral cortex**  **(µg/g brain)*^a^*** | **Cerebellum**  **(µg/g brain)*^a^*** |
| --- | --- | --- |
| hTfRMAb-1 | 0.776 | 0.992 |
| hTfRMAb-2 | 0.178 | 0.146 |
| hTfRMAb-3 | 0.673 | 0.827 |
| hTfRMAb-4 | 0.716 | 0.599 |

*^a^*, The data were previously reported in reference [2, (this is also cited as Ref. #28 in the main text)].

1. Fukatsu T, et al. Evaluation of brain delivery of blood-brain barrier-penetrable anti-human transferrin receptor monoclonal antibodies in monkeys. Experimental Animals. 2025;74(Suppl):102. (Meeting abstract)

| **Antibody** | **Pre-labeled EC_50_ values (ng/mL)^a^** | **Post-labeled EC_50_ values (ng/mL)^b^** |
| --- | --- | --- |
| hTfRMAb-1 | 31.57 | 66.76 |
| hTfRMAb-2 | 10.89 | 20.46 |
| hTfRMAb-3 | 10.33 | 48.03 |
| hTfRMAb-4 | 16.02 | 21.80 |

**Table S3. EC_50_ values of pre- and post-labeled hTfRMAbs binding to human TfR**

*^a^*, The data were previously reported in reference [2], where native hTfRMAbs were used.

*^b^*, The data were obtained using fluorescently labeled hTfRMAbs.

1. Fukatsu T, et al. Evaluation of brain delivery of blood-brain barrier-penetrable anti-human transferrin receptor monoclonal antibodies in monkeys. Experimental Animals. 2025;74(Suppl):102. (Meeting abstract)

**Table S4. Kinetic parameters of hTfRMAb**s **binding to human TfR**

| **Antibody** | **K_on_ (1/Ms)*^a^*** | | **K_off_ (1/s)*^b^*** | | **K_D_ (M)** | |
| --- | --- | --- | --- | --- | --- | --- |
|  | **n = 1** | **n = 2** | **n = 1** | **n = 2** | **n = 1** | **n = 2** |
| hTfRMAb-1 | 7.07 × 10^5^ | 7.09 × 10^-5^ | 3.24 × 10^-5^ | 8.13 × 10^-5^ | 4.58 × 10^-11^ | 1.15 × 10^-10^ |
| hTfRMAb-2 | 5.78 × 10^5^ | 4.91 × 10^-5^ | <1.0 × 10^-7^ | <1.0 × 10^-7^ | <1.0 × 10^-12^ | <1.0 × 10^-12^ |
| hTfRMAb-3 | 7.98 × 10^5^ | 6.99 × 10^-5^ | <1.0 × 10^-7^ | <1.0 × 10^-7^ | <1.0 × 10^-12^ | <1.0 × 10^-12^ |
| hTfRMAb-4 | 7.87 × 10^5^ | 8.76 × 10^-5^ | <1.0 × 10^-7^ | <1.0 × 10^-7^ | <1.0 × 10^-12^ | <1.0 × 10^-12^ |

*^a^*, k_on_, association rate constant.

*^b^*, k_off_, dissociation rate constant.

3. Supplementary materials: Figures and figure legends


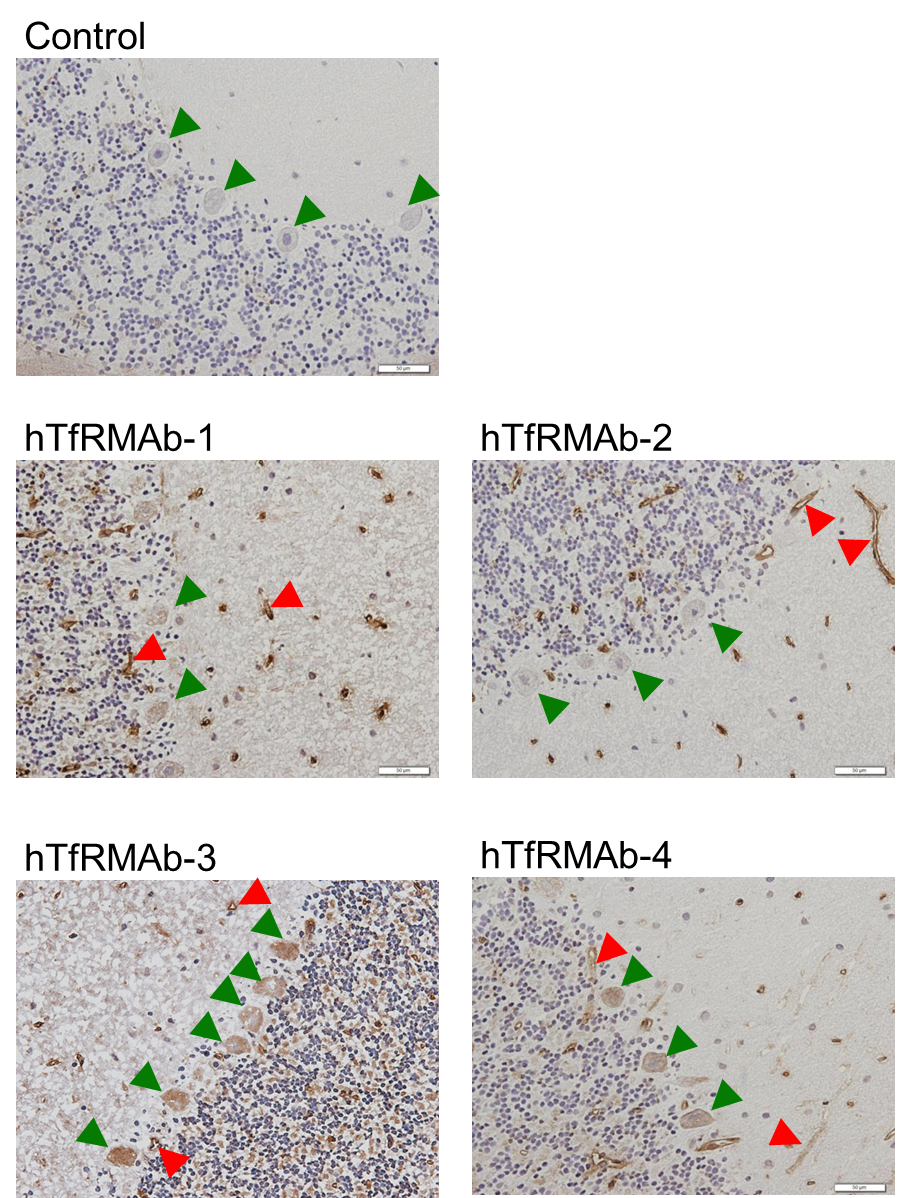


**Supplementary Figure S1. *In vivo* brain permeability of hTfRMAbs in cynomolgus monkeys.**

Immunohistochemical staining was performed to evaluate the distribution of each hTfRMAb in the cerebellum of cynomolgus monkeys at 8 hours post-administration. Brown staining indicates the presence of hTfRMAbs. Control represents a vehicle-treated animal. Notably, in all hTfRMAbs except for hTfRMAb-2, Purkinje cells were stained brown (green arrows), indicating that these three hTfRMAbs could reach the brain parenchyma following intravenous administration. In contrast, for hTfRMab-2, brown staining was observed only in blood vessels (red arrows), suggesting that it did not penetrate the BBB but rather remained confined within the vessel walls. Scale bar, 50 µm.

**Supplementary Figure S2. Uncropped Western blot images corresponding to Figure 1A.**

Full, uncropped images of Western blotting analyses (Figure 1A) detecting TfR and β-actin proteins are shown.
